# Exploring the Mechanisms of Gastrointestinal Cancer Development Using Deep Sequencing Analysis

**DOI:** 10.3390/cancers7020823

**Published:** 2015-06-15

**Authors:** Tomonori Matsumoto, Takahiro Shimizu, Atsushi Takai, Hiroyuki Marusawa

**Affiliations:** Department of Gastroenterology and Hepatology, Graduate School of Medicine, Kyoto University, 54 Shogoin-Kawahara-cho, Sakyo-ku, Kyoto 606-8507, Japan; E-Mails: tommatsu@kuhp.kyoto-u.ac.jp (T.M.); shimy@kuhp.kyoto-u.ac.jp (T.S.); atsushit@kuhp.kyoto-u.ac.jp (A.T.)

**Keywords:** mutational signature, deep sequencing, next-generation sequencing, inflammation-associated carcinogenesis, activation-induced cytidine deaminase, AID, APOBEC, transition, transversion, noncancerous tissues

## Abstract

Next-generation sequencing (NGS) technologies have revolutionized cancer genomics due to their high throughput sequencing capacity. Reports of the gene mutation profiles of various cancers by many researchers, including international cancer genome research consortia, have increased over recent years. In addition to detecting somatic mutations in tumor cells, NGS technologies enable us to approach the subject of carcinogenic mechanisms from new perspectives. Deep sequencing, a method of optimizing the high throughput capacity of NGS technologies, allows for the detection of genetic aberrations in small subsets of premalignant and/or tumor cells in noncancerous chronically inflamed tissues. Genome-wide NGS data also make it possible to clarify the mutational signatures of each cancer tissue by identifying the precise pattern of nucleotide alterations in the cancer genome, providing new information regarding the mechanisms of tumorigenesis. In this review, we highlight these new methods taking advantage of NGS technologies, and discuss our current understanding of carcinogenic mechanisms elucidated from such approaches.

## 1. Introduction

Recent innovations in next-generation sequencing (NGS) technologies have revolutionized cancer genome research due to their high throughput sequencing capacity. In fact, whole-exome sequencing (WES) and whole-genome sequencing (WGS) of various cancers using NGS technologies have led to the identification of many genetic alterations in cancerous tissues. Most of these genetic alterations might be passenger mutations that do not contribute to carcinogenesis, but some recurrently observed mutations are likely to be oncogenic driver mutations. Recent WES and/or WGS studies have uncovered some putative driver mutations by focusing on recurrently mutated genes among each cancer type. For example, WES analysis of glioblastoma multiformes led to the discovery of a previously unknown cancer-related gene, *isocitrate dehydrogenase 1* (*IDH1*) that is recurrently mutated in glioblastoma multiformes [1]. WES and/or WGS studies have also revealed particular profiles of genes mutated in each cancer [2]. In gastric cancers, frequent mutations of several cancer-related genes, such as *TP53*, *ARID1A*, and *CTNNB1*, were determined using WES and/or WGS [3,4,5,6,7]. Hepatocellular carcinomas also have mutations of genes in several pathways, including the p53/RB pathway (*TP53* and *CDKN2A*), WNT pathway (*CTNNB1* and *AXIN1*), and chromatin remodeling complex (*ARID1A* and *ARID2*) [8,9,10]. Thus, NGS technologies allowing for the accumulation of genome-wide sequencing data have elucidated driver mutation profiles of various cancers in the field of cancer genomics.

NGS technologies have also enabled us to approach the subject of carcinogenic mechanisms from new perspectives. Noncancerous tissues in premalignant conditions accumulate genetic aberrations before apparent tumor development, but such genetic aberrations often exist only in low allele frequencies and are thus difficult to detect by the classical Sanger sequencing method [11,12]. NGS technologies applied to read selected target regions can generate high depth data and reveal gene alterations with low allele frequencies. Identifying somatic mutations contained in noncancerous tissues by such (ultra-) deep sequencing could provide clues to elucidating carcinogenic mechanisms because those mutations might contribute to carcinogenesis at an early stage of tumor development. Moreover, analyses of the entire picture of mutations accumulated in cancerous tissues revealed that each cancer has particular mutational signatures reflecting its mutagenic mechanisms [13,14,15]. Thus, analysis of mutation profiles obtained by NGS and identification of the characteristics of mutational signatures of the cancers will lead to a better understanding of the underlying mutagenic processes. This review focuses on NGS approaches to explore carcinogenic mechanisms and the current knowledge revealed by recent studies.

## 2. Deep Sequencing of Premalignant Inflamed Tissues Using NGS Technologies

### 2.1. Importance of Studying Genetic Alterations in Inflamed Noncancerous Tissues

Many clinical, epidemiological, and biological studies have demonstrated that various factors trigger cancer development. Well-established mutagens, smoking and ultraviolet light, cause lung cancers and skin cancers by inducing genetic aberrations, respectively [16,17,18]. Germline mutations of some tumor suppressor genes strongly predispose to tumor development, as seen in familial polyposis coli and Li-Fraumeni syndrome [19]. Chronic inflammation also predisposes to inflammation-associated cancer development [20,21,22]. Especially in the gastrointestinal system, inflammation-associated cancers develop in various organs; chronic gastritis due to *Helicobacter pylori* (*H. pylori*) infection causes gastric cancers; chronic hepatic inflammation with hepatitis C virus (HCV) causes hepatocellular carcinoma; ulcerative colitis causes colitic cancers; and chronic duodenogastro-esophageal reflux and the resultant inflammation cause Barrett’s esophagus and esophageal adenocarcinoma [21]. Epithelial cells exposed to long-term inflammation have a strong potential for cancer development.

**Figure 1:**
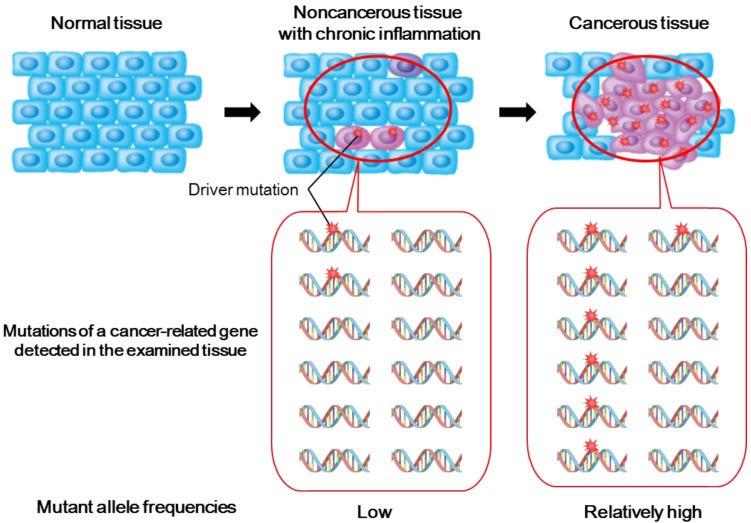
Scheme of accumulation of genetic alterations during inflammation-associated cancer development. Noncancerous tissues underlying chronic inflammation contain low-abundance mutated cells whose mutations can be detected as genetic alterations with low allele frequencies by (ultra-) deep sequencing. In contrast, cancerous tissues bear numbers of genetic aberrations including driver mutations (thorn-shaped), which are identified as mutations with relatively higher allele frequencies than those observed in noncancerous tissues. Some driver mutations in cancer-related genes may be shared between cancerous tissues and their underlying noncancerous tissues.

Cancer is a genome disease, and the accumulation of genetic aberrations in tumor-related genes is a critical step in malignant transformation [23]. Genetic aberrations that occur during cancer development accumulate in a stepwise manner, and mutations evoked by inflammation can be observed in inflamed noncancerous tissues even before tumor development (Figure 1) [11,21,22,24,25,26,27]. Thus, it is reasonable to assume that chronically inflamed epithelial cells play a role as the origin of inflammation-associated cancers through the accumulation of genetic alterations.

Recent NGS studies identified numerous genetic alterations in cancerous tissues, most of which are considered passenger mutations and only two to six of which are driver mutations that play a key role in cancer development [28]. It is possible that mutations latently accumulated in premalignant tissues also include putative driver mutations, which could contribute to the early stage of carcinogenesis. Therefore, studying genetic aberrations in noncancerous tissues could be a useful method for elucidating multistep carcinogenesis processes during inflammation-associated cancer development.

### 2.2. Genetic Alterations in Inflamed Noncancerous Tissues Determined by Conventional Sanger Sequencing

Several studies using the conventional Sanger sequencing method elucidated that noncancerous tissues at risk for inflammation-associated carcinogenesis contain somatic mutations in cancer-related genes. For example, Barrett’s esophagus epithelium represents premalignant lesions of esophageal adenocarcinoma with somatic mutations in *TP53* and *CDKN2A* genes, both of which are key tumor suppressor genes involved in the development of esophageal adenocarcinoma [11]. Chronic hepatitis tissues with HCV infection, which predisposes to hepatocellular carcinoma, bear *TP53* mutations at frequencies of 4–15 nucleotides per 10^4^ nucleotides [26]. A sequencing study of colon crypts isolated by laser capture microdissection revealed *TP53* mutations in both premalignant dysplasia and nondysplastic inflamed colon crypts of patients with ulcerative colitis [12]. These findings suggest that chronically inflamed epithelia sustain founder mutations for carcinogenesis before malignant transformation. Because of the low frequencies of cells with mutated genes in noncancerous tissues (Figure 1), however, identifying pro-oncogenic mutations in noncancerous tissues by the Sanger method requires much time and effort, and is still insufficient.

### 2.3. Deep Sequencing Analysis of Inflamed Noncancerous Tissues

Deep or ultradeep sequencing using NGS technologies, which often read each nucleotide more than thousands of times, is an efficient tool for the detection of genetic alterations with very low frequencies of mutated alleles [29]. For example, mutated alleles with frequencies as low as 1% or possibly even 0.1% can be detected by deep sequencing under conditions in which the error rate during the sequencing process is properly evaluated and suppressed [7,29]. Taking advantage of the high sensitivity of NGS for the detection of low-abundance mutations, recent studies identified somatic mutations of cancer-related genes in noncancerous tissues with chronic inflammation in various organs.

*TP53* mutations are most frequently detected in gastric cancer genomes, followed by *ARID1A*, *CTNNB1*, and *PIK3CA* [3,4,30]. We performed deep sequencing on selected tumor-related genes in *H. pylori*-related severe gastritis mucosa, which is considered to be a high-risk condition for gastric cancer [7]. In the gastritis mucosa of 28 patients with gastric cancer, non-synonymous low-abundance mutations in *TP53* and *ARID1A* were detected in 11 cases (39.3%) and four cases (14.3%), respectively. Interestingly, non-synonymous low-abundance mutations in *TP53* and *ARID1A* were also detected in the gastritis mucosa of patients without gastric cancer [7]. These findings suggest that various mutations in tumor-related genes latently accumulate in *H. pylori*-related gastritis mucosa before histological malignant changes.

Consistent with our findings, other recent studies revealed that the majority of recurrent mutations in cancer-related genes detected in esophageal adenocarcinoma were found in Barrett’s esophagus [31,32,33]. Weaver *et al.* performed targeted deep sequencing analyses on esophageal adenocarcinoma tissues and on benign metaplastic never-dysplastic Barrett’s esophagus and high-grade dysplasia, which are two key transition points in the development from premalignant Barrett’s esophagus to esophageal adenocarcinoma [33]. Somatic mutations in 26 analyzed cancer-related genes were detected in 53% of individuals with never-dysplastic Barrett’s esophagus and in 91% of those with high-grade dysplasia. Furthermore, the vast majority of detected mutations had similar mutation frequencies among the three disease stages, while *TP53* was mutated exclusively in high-grade dysplasia and esophageal adenocarcinoma, but not in never-dysplastic Barrett’s esophagus. Combined with accessional analyses, the *TP53* mutation status was found to differentiate never-dysplastic Barrett’s esophagus from high-grade dysplasia and esophageal adenocarcinoma [33].

With regard to hepatobiliary tumors, we demonstrated that many genetic alterations accumulate in the cirrhotic liver following HCV-related chronic hepatitis, a predisposing condition to hepatocellular carcinoma [34]. Whole exome sequencing on nontumorous cirrhotic liver tissues led to the identification of nucleotide alterations in a large quantity, comparable to those of hepatocellular carcinoma, while the mutation frequencies in cirrhotic tissues tended to be lower than those in the matched tumor tissues. Although the majority of the mutated genes detected in cirrhotic tissues were thought to be passenger mutations, the leptin receptor gene (*LEPR*) was identified as a putative cancer-related gene mutated in both cirrhotic tissues and hepatocellular carcinoma. Additional deep sequencing analyses on *TP53*, *CTNNB1*, and *LEPR* genes revealed low-abundance mutations in more than half of the nontumorous cirrhotic tissues analyzed. In addition, Jiang *et al.* reported whole exome sequencing on one dysplastic nodule and two hepatocellular carcinomas in the same patient with HBV infection [35], and, consistent with our results, several mutations were detected in dysplastic nodules as well as tumor tissues, although there was no overlap in the mutations between dysplastic nodules and hepatocellular carcinomas. These findings indicate that oncogenic mutations of genes related to hepatocarcinogenesis latently accumulate in cirrhotic livers with viral infection.

Thus, deep sequencing analyses on inflamed noncancerous tissues have elucidated the accumulation of putative pro-oncogenic mutations of cancer-related genes in the noncancerous tissues of various organs during the process of inflammation-associated carcinogenesis.

## 3. Mutation Signatures Provide Clues to Predict Tumorigenic Mechanisms

### 3.1. Mutational Signatures Specific to Various Types of Cancers

Based on genome-wide mutation profiles of various cancers revealed by NGS technologies, each cancer has a unique mutational signature [13,14,15]. Mutational signatures are classified according to the type of mutations, such as substitution and small insertions and deletions (indels), and the sequence contexts of the mutations [13]. With regard to single nucleotide substitutions, all substitutions can be classified into 96 patterns by six patterns of base substitution (C:G>T:A, C:G>A:T, C:G>G:C, T:A>C:G, T:A>A:T, T:A>G:C) and the bases immediately 5ʹ and 3ʹ to each substitution. This 96-substitution classification is intelligible and well analyzed in many studies [13,36,37].

**Figure 2:**
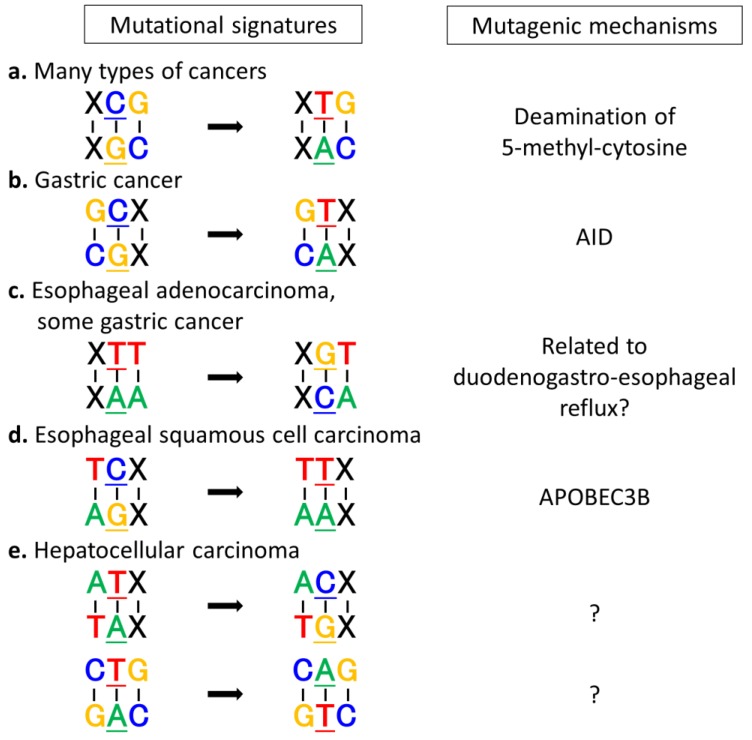
Dominant mutational signatures in gastrointestinal cancers and their putative contributors. (**a**) C:G>T:A transitions at XpCpG trinucleotides are the most prominent mutational signature in many types of cancers, including gastric cancer, esophageal squamous cell carcinoma, esophageal adenocarcinoma, and hepatocellular carcinoma, and are considered to be related to spontaneous deamination of 5-methylcytosine; (**b**) C:G>T:A transitions at GpCpX sequences, which are dominantly observed in gastric cancers, accord with representative footprints of AID-mediated cytidine deamination; (**c**) T:A>G:C transversions at XpTpT sites are uniquely observed in esophageal adenocarcinomas and some microsatellite-stable gastric cancers, suggesting that some mutagenic processes related to duodenogastro-esophageal reflux are shared between these cancers; (**d**) C:G>T:A transitions at TpCpX sequences suggest the involvement of APOBEC3B in the development of esophageal squamous cell carcinoma; (**e**) T:A>C:G transitions in ApTpX sites and T:A>A:T transversions in CpTpG sites are characteristically detected in hepatocellular carcinoma, whose causes remain to be determined.

Several studies clarified that C:G>T:A transitions at XpCpG trinucleotides (X: any nucleotide, under bar: mutated nucleotide) are the most prominent mutational signature in many types of cancers, particularly gastrointestinal cancers (Figure 2) [13,14,36]. In addition, each type of cancer has its own specific dominant mutational signature. In gastric cancer, C:G>T:A transitions at XpCpG sites as well as GpCpX sites are dominantly observed [6,7], and high rates of T:A>G:C transversions at XpTpT sites (specifically at CpTpT sites) were also recently observed in some microsatellite-stable gastric cancers [30,38]. C:G>T:A transitions at GpCpX sites and T:A>G:C transversions at XpTpT sites are unique patterns of gastric cancer genomes. Esophageal cancers have two different histological types: adenocarcinoma caused by duodenogastro-esophageal reflux, and squamous cell carcinoma, whose risk factors are tobacco and alcohol. While T:A>G:C transversions at XpTpT sites are most frequently observed in esophageal adenocarcinomas similar to some gastric cancers [33,39], C:G>T:A transitions at XpCpG trinucleotides are the most predominant patterns in esophageal squamous cell carcinoma, followed by C:G>G:C transversions and C:G>T:A transitions at TpCpX motifs [40,41,42]. Hepatocellular carcinoma has unique mutational signatures, such as T:A>C:G transitions in ApTpX sequences and T:A>A:T transversions in CpTpG sequences, in addition to C:G>T:A transitions in XpCpG motifs [9,43,44]. Interestingly, cholangiocarcinoma, another histological type of liver tumor, does not have these characteristic mutational signatures [45,46]. In colorectal cancer and pancreatic cancer, no specific mutational patterns have been detected, although some tumors with defective DNA repair genes, including DNA mismatch repair genes, DNA polymerase genes, or *BRCA1/2*, exhibit characteristic patterns [13]. These findings indicate that tumor mutational signatures differ based on their origin.

### 3.2. Extrinsic and Intrinsic Mutagens and Mutational Signatures

Mutational signatures that accumulate in the cancer genome provide clues to identifying the cause of genetic alterations during tumor development because many mutagenic agents and repair processes have biased mutational patterns and preferred target nucleotide sequences [13,37,47,48]. For example, loss of function in DNA repair genes induces specific patterns of genomic alterations. Tumors with microsatellite instability in many cancer types have numerous substitutions and indels due to defects of mismatch repair function caused by promoter methylation of *MLH1* or mutations of *MSH2*, *MSH3*, and *MSH6* [49,50,51]. Tumors with mutations in *POLE* or *POLD1* have extreme numbers of mutations due to an impaired proofreading function of DNA polymerases [52,53]. Some tumors with inactivating mutations of *BRCA1* or *BRCA2*, such as some breast and pancreatic cancers, have substantial numbers of larger deletions (up to 50 bp) with overlapping microhomology at breakpoint junctions [13,54]. By contrast, many toxigenic factors have been investigated as extrinsic mutagens. Ultraviolet light, a well-known extrinsic mutagen, mainly induces C:G>T:A transitions in dipyrimidines, and accordingly this mutation pattern is predominant in melanoma and basal cell carcinoma, providing evidence that ultraviolet light is a causative factor in the development of these tumors [55,56]. Benzo[a]pyrene, a convincingly established carcinogen contained in tobacco, is likely to cause C:G>A:T transversions, and this mutation pattern is dominantly observed in lung cancers, especially in those associated with smokers [13,57].

As for intrinsic mutagens, several studies have demonstrated that apolipoprotein B mRNA editing enzyme catalytic polypeptide (APOBEC) family members play an important role in the development of various cancers by inducing genetic aberrations via their nucleotide-editing activity [36,58]. Among APOBEC family members, activation-induced cytidine deaminase (AID) was first characterized by its ability to induce genetic changes in genome DNA sequences and is considered to act as an intrinsic mutagen during the process of inflammation-associated carcinogenesis [21,59]. Under physiological conditions, AID is expressed almost exclusively in activated B lymphocytes, and contributes to generating antibody gene diversification by inducing somatic hypermutations and class-switch recombinations of immunoglobulin genes [60,61]. On the other hand, inflammatory stimulation elicits aberrant AID expression in epithelial cells and overexpressed AID could induce mutations in various non-immunoglobulin genes and trigger inflammation-associated tumorigenesis, including gastric carcinogenesis associated with *H. pylori*-related gastritis and hepatocarcinogenesis associated with chronic hepatitis C [26,62,63]. AID deaminates C to U, resulting in the generation of a U:G mismatch. This U:G mismatch is resolved by several pathways, such as the mismatch repair system. If the mismatch is not repaired before the onset of DNA replication, DNA polymerase will insert an A nucleotide opposite the U nucleotide, resulting in C:G>T:A transitions [64]. Such AID-induced mutagenesis is genome-widely confirmed by experimental models in which AID predominantly caused C:G>T:A transitions in the known preferred AID target sequence, *i.e.*, WRCY motifs (W = A or T, R = A or G, and Y = C or T) or RpCpX trinucleotides [47,65]. APOBEC3B, which is another member of APOBEC family, is an enzyme that can induce genomic alterations in human genomes [36,66]. In contrast to AID, APOBEC3B exhibits a strong preference for deaminating C residues flanked by T. Although the function of APOBEC3B in normal conditions is unknown, APOBEC3B expression is correlated with frequencies of C:G>T:A transitions or C:G>G:C transversions in TpCpX motifs in several types of cancer, including breast cancer and lung cancer [36,48]. In addition, foci of localized hypermutations with an APOBEC3B-mediated pattern, referred to as kataegis, are often seen in these cancers and are associated with genomic rearrangements [13,36,54]. These findings suggest that APOBEC3B is related to the generation of both mutations and chromosomal aberrations in these cancers. The mechanisms of APOBEC3B upregulation in these tissues, however, remain unknown.

### 3.3. Exploring Carcinogenic Mechanisms by Analyzing Mutational Signatures

Analysis of mutational signatures in cancers can be an effective method to explore carcinogenic mechanisms as dominant mutational signatures observed in each cancer possibly reflect the mutagenic mechanisms during carcinogenesis (Figure 2).

As for inflammation-associated cancers in gastrointestinal organs, AID is a promising key mutagen that contributes to tumorigenesis via its DNA-editing activity. We previously demonstrated that AID is aberrantly expressed in inflamed epithelial cells in various human organs [21,58], and AID transgenic mice with constitutive and ubiquitous AID expression develop gastric cancers and hepatocellular carcinomas via the mutagenic activity of AID [67]. Moreover, analyses of mutational signatures strongly support the hypothesis that AID is involved in the development of *H. pylori*-related gastric cancers. In gastric cancers, the most common mutation is C:G>T:A transitions, more than half of which occur in XpCpG trinucleotides [3,4]. Such transition at the XpCpG site is the prominent mutational signature in many types of cancer, and is considered to be related to spontaneous deamination of 5-methylcytosine [13,68]. In addition, gastric cancers also have a preponderance of C:G>T:A transitions at non-CpG sites, especially at GpCpX sequences [6,7]. Interestingly, this mutational pattern corresponds well with the mutational signature induced by AID activity, suggesting the involvement of AID-mediated cytidine deamination in the induction of somatic mutations during gastric carcinogenesis. Moreover, deep sequencing on selected cancer-related genes in nontumorous gastritis mucosa revealed a strong preference for C:G>T:A transitions at GpCpX sequences, similar to those in gastric cancer tissues [7]. These findings suggest that AID consistently contributes to the development of gastric cancer from the initiation stage to the promotion stage.

In esophageal adenocarcinomas, the predominant mutational signature is T:A>G:C transversions with striking enrichment at the CpTpT site [31,33,39]. This mutational signature is relatively rare in other cancers, but some microsatellite-stable gastric cancers exhibit the same pattern [38]. These findings suggest that some factors related to duodenogastro-esophageal reflux and its resultant inflammation or unknown mutagens are associated with carcinogenesis in these regions. The fact that the essential mutations in cancer-related genes, such as *TP53*, *CDKN2A*, *SMAD4* and *PIK3CA*, are not affected by these transversions at XpTpT sites, however, makes it difficult to understand key factors in the development of these cancers [39]. On the other hand, the predominant mutational signature of esophageal squamous cell carcinoma is C:G>T:A transitions at XpCpG and TpCpX sites, followed by C:G>G:C transversions [31,40,41,42]. C:G>T:A transitions at TpCpX sequences represent the involvement of APOBEC3B in the development of esophageal squamous cell carcinoma, consistent with the fact that APOBEC3B expression is upregulated in these tumors [41]. The cause of C:G>G:C transversions, however, remains unknown. Interestingly, C:G>A:T transversions, which are dominantly detected in lung squamous cell carcinoma, are not predominant patterns in esophageal squamous cell carcinoma [28,39]. These findings suggest that some mutagenic processes other than those associated with tobacco carcinogens are involved in the development of esophageal squamous cell carcinoma, although further investigation is needed.

The mutational patterns of hepatocellular carcinomas are quite different from those of gastrointestinal cancers, although both cancer types are deeply associated with chronic inflammation. In addition to C:G>T:A transitions in XpCpG contexts, T:A>C:G transitions in ApTpX contexts and T:A>A:T transversions in CpTpG contexts are characteristic patterns in hepatocellular carcinoma [9,13,43,44]. Recent reports indicate that these mutational patterns are associated with ancestry and sex, but not with viral status [44]. While C:G>T:A transitions in XpCpG contexts are commonly observed across all ancestry and sexes, T:A>C:G transitions in ApTpA contexts and T:A>A:T transversions in CpTpG contexts are especially increased in Japanese males and US-Asian cases, respectively [44]. These findings suggest that intra-ancestry diversity and/or environmental factors are associated with the development of hepatocellular carcinoma. Interestingly, these mutational signatures are strongly connected with transcriptional strand biases, suggesting the involvement of transcription-coupled DNA repair that operates predominantly on the transcribed strand of the genes [13,44]. Considering that transcription-coupled DNA repair generally works on bulky DNA helix-distorting lesions, unknown extrinsic mutagens may be related to hepatocellular carcinogenesis.

Although the mutation pattern of each cancer provides useful information about carcinogenic mechanisms, several kinds of mutational signatures often coexist in a particular cancer and characteristics of genetic alterations varies according to molecular and histological subtypes. For example, in gastric cancers, microsatellite-stable cancers exhibit chromosomal instability and T:A>G:C transversions at the CpTpT site; microsatellite-instable cancers have features of chromosomal stability and a large number of single nucleotide substitutions with relatively high T:A>C:G transition rates; diffuse-type cancers have relatively fewer single nucleotide variants and copy number aberrations [38]. Although subtyping of cancers by mutational signatures has not been established yet, it is expected that a cancer will be classified into some genetic subtypes based on mutational signatures and other characteristics of genetic aberrations in addition to traditional classification.

## 4. Conclusions

NGS technologies have uncovered not only genetic alterations of tumor tissues, but also those with low allele frequencies in noncancerous tissues. Moreover, mutational signatures determined by NGS also provide the footprints of carcinogenic processes. Interpreting mutational signatures of noncancerous tissues in combination with those of cancerous tissues will provide information about the processes of initiation and promotion of various cancers. There are various patterns of mutational signatures observed in human cancers whose mutagenic processes are not yet explained. Resolution of these remaining mysteries will be helpful for elucidating carcinogenic mechanisms.

NGS technologies have also exerted a great effect on identification of major driver genes in various cancers, however, to reveal rare remaining driver mutations may be difficult due to limits of sample sizes [69]. On the other hand, biomarkers overexpressed in cancers could derive from a small proportion of tumor cells which possess a certain genetic alteration. Thus, the NGS technologies which provide high sequencing coverage can be a powerful tool to identify such genetic aberrations and the resultant overexpression of biomarker proteins.

## References

[1] Parsons D.W., Jones S., Zhang X., Lin J.C., Leary R.J., Angenendt P., Mankoo P., Carter H., Siu I., Gallia G.L. (2008). An integrated genomic analysis of human glioblastoma multiforme. Science.

[2] Watson I.R., Takahashi K., Futreal P.A., Chin L. (2013). Emerging patterns of somatic mutations in cancer. Nat. Rev. Genet..

[3] Wang K., Kan J., Yuen S.T., Shi S.T., Chu K.M., Law S., Chan T.L., Kan Z., Chan A.S.Y., Tsui W.Y. (2011). Exome sequencing identifies frequent mutation of ARID1A in molecular subtypes of gastric cancer. Nat. Genet..

[4] Zang Z.J., Cutcutache I., Poon S.L., Zhang S.L., McPherson J.R., Tao J., Rajasegaran V., Heng H.L., Deng N., Gan A. (2012). Exome sequencing of gastric adenocarcinoma identifies recurrent somatic mutations in cell adhesion and chromatin remodeling genes. Nat. Genet..

[5] Wang K., Yuen S.T., Xu J., Lee S.P., Yan H.H.N., Shi S.T., Siu H.C., Deng S., Chu K.M., Law S. (2014). Whole-genome sequencing and comprehensive molecular profiling identify new driver mutations in gastric cancer. Nat. Genet..

[6] Nagarajan N., Bertrand D., Hillmer A.M., Zang Z.J., Yao F., Jacques P.-É., Teo A.S.M., Cutcutache I., Zhang Z., Lee W.H. (2012). Whole-genome reconstruction and mutational signatures in gastric cancer. Genome Biol..

[7] Shimizu T., Marusawa H., Matsumoto Y., Inuzuka T., Ikeda A., Fujii Y., Minamiguchi S., Miyamoto S., Kou T., Sakai Y. (2014). Accumulation of somatic mutations in TP53 in gastric epithelium with *Helicobacter pylori* infection. Gastroenterology.

[8] Guichard C., Amaddeo G., Imbeaud S., Ladeiro Y., Pelletier L., Maad I.B., Calderaro J., Bioulac-Sage P., Letexier M., Degos F. (2012). Integrated analysis of somatic mutations and focal copy-number changes identifies key genes and pathways in hepatocellular carcinoma. Nat. Genet..

[9] Fujimoto A., Totoki Y., Abe T., Boroevich K.A., Hosoda F., Nguyen H.H., Aoki M., Hosono N., Kubo M., Miya F. (2012). Whole-genome sequencing of liver cancers identifies etiological influences on mutation patterns and recurrent mutations in chromatin regulators. Nat. Genet..

[10] Nakagawa H., Shibata T. (2013). Comprehensive genome sequencing of the liver cancer genome. Cancer Lett..

[11] Barrett M.T., Sanchez C.A., Prevo L.J., Wong D.J., Galipeau P.C., Paulson T.G., Rabinovitch P.S., Reid B.J. (1999). Evolution of neoplastic cell lineages in Barrett oesophagus. Nat. Genet..

[12] Leedham S.J., Graham T.A., Oukrif D., McDonald S.A.C., Rodriguez-Justo M., Harrison R.F., Shepherd N.A., Novelli M.R., Jankowski J.A.Z., Wright N.A. (2009). Clonality, founder mutations, and field cancerization in human ulcerative colitis-associated neoplasia. Gastroenterology.

[13] Alexandrov L.B., Nik-Zainal S., Wedge D.C., Aparicio S.A.J.R., Behjati S., Biankin A.V., Bignell G.R., Bolli N., Borg A., Børresen-Dale A.-L. (2013). Signatures of mutational processes in human cancer. Nature.

[14] Lawrence M.S., Stojanov P., Polak P., Kryukov G.V., Cibulskis K., Sivachenko A., Carter S.L., Stewart C., Mermel C.H., Roberts S.A. (2013). Mutational heterogeneity in cancer and the search for new cancer-associated genes. Nature.

[15] Greenman C., Stephens P., Smith R., Dalgliesh G.L., Hunter C., Bignell G., Davies H., Teague J., Butler A., Stevens C. (2007). Patterns of somatic mutation in human cancer genomes. Nature.

[16] Hecht S.S. (2003). Tobacco carcinogens, their biomarkers and tobacco-induced cancer. Nat. Rev. Cancer.

[17] Brash D.E., Rudolph J.A., Simon J.A., Lin A., McKenna G.J., Baden H.P., Halperin A.J., Pontén J. (1991). A role for sunlight in skin cancer: UV-induced p53 mutations in squamous cell carcinoma. Proc. Natl. Acad. Sci. USA.

[18] Pfeifer G.P., You Y.-H., Besaratinia A. (2005). Mutations induced by ultraviolet light. Mutat. Res..

[19] Foulkes W.D. (2008). Inherited susceptibility to common cancers. N. Engl. J. Med..

[20] Coussens L.M., Werb Z. (2002). Inflammation and cancer. Nature.

[21] Chiba T., Marusawa H., Ushijima T. (2012). Inflammation-associated cancer development in digestive organs: Mechanisms and roles for genetic and epigenetic modulation. Gastroenterology.

[22] Colotta F., Allavena P., Sica A., Garlanda C., Mantovani A. (2009). Cancer-related inflammation, the seventh hallmark of cancer: Links to genetic instability. Carcinogenesis.

[23] Hanahan D., Weinberg R.A. (2011). Hallmarks of cancer: The next generation. Cell.

[24] Brentnall T.A., Haggitt R.C., Rabinovitch P.S., Kimmey M.B., Bronner M.P., Levine D.S., Kowdley K.V., Stevens A.C., Crispin D.A., Emond M. (1996). Risk and natural history of colonic neoplasia in patients with primary sclerosing cholangitis and ulcerative colitis. Gastroenterology.

[25] Hussain S.P., Amstad P., Raja K., Ambs S., Nagashima M., Bennett W.P., Shields P.G., Ham A.J., Swenberg J.A., Marrogi A.J. (2000). Increased p53 mutation load in noncancerous colon tissue from ulcerative colitis: A cancer-prone chronic inflammatory disease. Cancer Res..

[26] Kou T., Marusawa H., Kinoshita K., Endo Y., Okazaki I.-M., Ueda Y., Kodama Y., Haga H., Ikai I., Chiba T. (2007). Expression of activation-induced cytidine deaminase in human hepatocytes during hepatocarcinogenesis. Int. J. Cancer.

[27] Hamada S., Masamune A., Shimosegawa T. (2014). Inflammation and pancreatic cancer: Disease promoter and new therapeutic target. J. Gastroenterol..

[28] Kandoth C., McLellan M.D., Vandin F., Ye K., Niu B., Lu C., Xie M., Zhang Q., McMichael J.F., Wyczalkowski M.A. (2013). Mutational landscape and significance across 12 major cancer types. Nature.

[29] Yoshida K., Sanada M., Ogawa S. (2013). Deep sequencing in cancer research. Jpn. J. Clin. Oncol..

[30] Bass A.J., Thorsson V., Shmulevich I., Reynolds S.M., Miller M., Bernard B., Hinoue T., Laird P.W., Curtis C., Shen H. (2014). Comprehensive molecular characterization of gastric adenocarcinoma. Nature.

[31] Agrawal N., Jiao Y., Bettegowda C., Hutfless S.M., Wang Y., David S., Cheng Y., Twaddell W.S., Latt N.L., Shin E.J. (2012). Comparative genomic analysis of esophageal adenocarcinoma and squamous cell carcinoma. Cancer Discov..

[32] Streppel M.M., Lata S., Delabastide M., Montgomery E.A., Wang J.S., Canto M.I., Macgregor-Das A.M., Pai S., Morsink F.H.M., Offerhaus G.J. (2014). Next-generation sequencing of endoscopic biopsies identifies ARID1A as a tumor-suppressor gene in Barrett’s esophagus. Oncogene.

[33] Weaver J.M.J., Ross-Innes C.S., Shannon N., Lynch A.G., Forshew T., Barbera M., Murtaza M., Ong C.-A.J., Lao-Sirieix P., Dunning M.J. (2014). Ordering of mutations in preinvasive disease stages of esophageal carcinogenesis. Nat. Genet..

[34] Ikeda A., Shimizu T., Matsumoto Y., Fujii Y., Eso Y., Inuzuka T., Mizuguchi A., Shimizu K., Hatano E., Uemoto S. (2014). Leptin receptor somatic mutations are frequent in HCV-infected cirrhotic liver and associated with hepatocellular carcinoma. Gastroenterology.

[35] Jiang J.H., Liu Y.F., Ke A.W., Gu F.M., Yu Y., Dai Z., Gao Q., Shi G.M., Liao B.Y., Xie Y.H. (2014). Clinical significance of the ubiquitin ligase UBE3C in hepatocellular carcinoma revealed by exome sequencing. Hepatology.

[36] Burns M.B., Temiz N.A., Harris R.S. (2013). Evidence for APOBEC3B mutagenesis in multiple human cancers. Nat. Genet..

[37] Poon S., McPherson J.R., Tan P., Teh B., Rozen S.G. (2014). Mutation signatures of carcinogen exposure: Genome-wide detection and new opportunities for cancer prevention. Genome Med..

[38] Wang K., Yuen S.T., Xu J., Lee S.P., Yan H.H.N., Shi S.T., Siu H.C., Deng S., Chu K.M., Law S. (2014). Whole-genome sequencing and comprehensive molecular profiling identify new driver mutations in gastric cancer. Nat. Genet..

[39] Dulak A.M., Stojanov P., Peng S., Lawrence M.S., Fox C., Stewart C., Bandla S., Imamura Y., Schumacher S.E., Shefler E. (2013). Exome and whole-genome sequencing of esophageal adenocarcinoma identifies recurrent driver events and mutational complexity. Nat. Genet..

[40] Gao Y., Chen Z., Li J., Hu X., Shi X., Sun Z., Zhang F., Zhao Z., Li Z., Liu Z. (2014). Genetic landscape of esophageal squamous cell carcinoma. Nat. Genet..

[41] Lin D.-C., Hao J.-J., Nagata Y., Xu L., Shang L., Meng X., Sato Y., Okuno Y., Varela A.M., Ding L.-W. (2014). Genomic and molecular characterization of esophageal squamous cell carcinoma. Nat. Genet..

[42] Song Y., Li L., Ou Y., Gao Z., Li E., Li X., Zhang W., Wang J., Xu L., Zhou Y. (2014). Identification of genomic alterations in oesophageal squamous cell cancer. Nature.

[43] Totoki Y., Tatsuno K., Yamamoto S., Arai Y., Hosoda F., Ishikawa S., Tsutsumi S., Sonoda K., Totsuka H., Shirakihara T. (2011). High-resolution characterization of a hepatocellular carcinoma genome. Nat. Genet..

[44] Totoki Y., Tatsuno K., Covington K.R., Ueda H., Creighton C.J., Kato M., Tsuji S., Donehower L.A., Slagle B.L., Nakamura H. (2014). Trans-ancestry mutational landscape of hepatocellular carcinoma genomes. Nat. Genet..

[45] Chan-On W., Nairismägi M.-L., Ong C.K., Lim W.K., Dima S., Pairojkul C., Lim K.H., McPherson J.R., Cutcutache I., Heng H.L. (2013). Exome sequencing identifies distinct mutational patterns in liver fluke-related and non-infection-related bile duct cancers. Nat. Genet..

[46] Jiao Y., Pawlik T.M., Anders R.A., Selaru F.M., Streppel M.M., Lucas D.J., Niknafs N., Guthrie V.B., Maitra A., Argani P. (2013). Exome sequencing identifies frequent inactivating mutations in BAP1, ARID1A and PBRM1 in intrahepatic cholangiocarcinomas. Nat. Genet..

[47] Olivier M., Weninger A., Ardin M., Huskova H., Castells X., Vallée M.P., McKay J., Nedelko T., Muehlbauer K.-R., Marusawa H. (2014). Modelling mutational landscapes of human cancers *in vitro*. Sci. Rep..

[48] Roberts S.A., Lawrence M.S., Klimczak L.J., Grimm S.A., Fargo D., Stojanov P., Kiezun A., Kryukov G.V., Carter S.L., Saksena G. (2013). An APOBEC cytidine deaminase mutagenesis pattern is widespread in human cancers. Nat. Genet..

[49] Peltomäki P. (2003). Role of DNA mismatch repair defects in the pathogenesis of human cancer. J. Clin. Oncol..

[50] Imai K., Yamamoto H. (2008). Carcinogenesis and microsatellite instability: The interrelationship between genetics and epigenetics. Carcinogenesis.

[51] Shah S.N., Hile S.E., Eckert K.A. (2010). Defective mismatch repair, microsatellite mutation bias, and variability in clinical cancer phenotypes. Cancer Res..

[52] Heitzer E., Tomlinson I. (2014). Replicative DNA polymerase mutations in cancer. Curr. Opin. Genet. Dev..

[53] Palles C., Cazier J.-B., Howarth K.M., Domingo E., Jones A.M., Broderick P., Kemp Z., Spain S.L., Guarino E., Guarino Almeida E. (2013). Germline mutations affecting the proofreading domains of POLE and POLD1 predispose to colorectal adenomas and carcinomas. Nat. Genet..

[54] Nik-Zainal S., Alexandrov L.B., Wedge D.C., van Loo P., Greenman C.D., Raine K., Jones D., Hinton J., Marshall J., Stebbings L.A. (2012). Mutational processes molding the genomes of 21 breast cancers. Cell.

[55] Krauthammer M., Kong Y., Ha B.H., Evans P., Bacchiocchi A., McCusker J.P., Cheng E., Davis M.J., Goh G., Choi M. (2012). Exome sequencing identifies recurrent somatic RAC1 mutations in melanoma. Nat. Genet..

[56] Jayaraman S.S., Rayhan D.J., Hazany S., Kolodney M.S. (2014). Mutational landscape of basal cell carcinomas by whole-exome sequencing. J. Invest. Dermatol..

[57] Pfeifer G.P., Denissenko M.F., Olivier M., Tretyakova N., Hecht S.S., Hainaut P. (2002). Tobacco smoke carcinogens, DNA damage and p53 mutations in smoking-associated cancers. Oncogene.

[58] Shimizu T., Marusawa H., Endo Y., Chiba T. (2012). Inflammation-mediated genomic instability: Roles of activation-induced cytidine deaminase in carcinogenesis. Cancer Sci..

[59] Takai A., Marusawa H., Chiba T. (2011). Acquisition of Genetic Aberrations by Activation-Induced Cytidine Deaminase (AID) during Inflammation-Associated Carcinogenesis. Cancers.

[60] Muramatsu M., Kinoshita K., Fagarasan S., Yamada S., Shinkai Y., Honjo T. (2000). Class switch recombination and hypermutation require activation-induced cytidine deaminase (AID), a potential RNA editing enzyme. Cell.

[61] Honjo T., Kinoshita K., Muramatsu M. (2002). Molecular mechanism of class switch recombination: Linkage with somatic hypermutation. Annu. Rev. Immunol..

[62] Matsumoto Y., Marusawa H., Kinoshita K., Endo Y., Kou T., Morisawa T., Azuma T., Okazaki I.-M., Honjo T., Chiba T. (2007). Helicobacter pylori infection triggers aberrant expression of activation-induced cytidine deaminase in gastric epithelium. Nat. Med..

[63] Endo Y., Marusawa H., Kinoshita K., Morisawa T., Sakurai T., Okazaki I.-M., Watashi K., Shimotohno K., Honjo T., Chiba T. (2007). Expression of activation-induced cytidine deaminase in human hepatocytes via NF-kappaB signaling. Oncogene.

[64] Liu M., Schatz D.G. (2009). Balancing AID and DNA repair during somatic hypermutation. Trends Immunol..

[65] Kim S.K., Nasu A., Komori J., Shimizu T., Matsumoto Y., Minaki Y., Kohno K., Shimizu K., Uemoto S., Chiba T. (2014). A model of liver carcinogenesis originating from hepatic progenitor cells with accumulation of genetic alterations. Int. J. Cancer.

[66] Shinohara M., Io K., Shindo K., Matsui M., Sakamoto T., Tada K., Kobayashi M., Kadowaki N., Takaori-Kondo A. (2012). APOBEC3B can impair genomic stability by inducing base substitutions in genomic DNA in human cells. Sci. Rep..

[67] Morisawa T., Marusawa H., Ueda Y., Iwai A., Okazaki I., Honjo T., Chiba T. (2008). Organ-specific profiles of genetic changes in cancers caused by activation-induced cytidine deaminase expression. Int. J. Cancer.

[68] Shen J.C., Rideout W.M., Jones P.A. (1994). The rate of hydrolytic deamination of 5-methylcytosine in double-stranded DNA. Nucleic Acids Res..

[69] Wood A.R., Tuke M.A., Nalls M., Hernandez D., Gibbs J.R., Lin H., Xu C.S., Li Q., Shen J., Jun G. (2014). Whole-genome sequencing to understand the genetic architecture of common gene expression and biomarker phenotypes. Hum. Mol. Genet..

